# Maturation of auditory temporal integration and inhibition assessed with event-related potentials (ERPs)

**DOI:** 10.1186/1471-2202-11-49

**Published:** 2010-04-16

**Authors:** Allison M Fox, Mike Anderson, Corinne Reid, Tim Smith, Dorothy VM Bishop

**Affiliations:** 1 School of Psychology, University of Western Australia, Perth, Australia; 2 School of Psychology, Murdoch University, Perth, Australia; 3 Department of Experimental Psychology, University of Oxford, Oxford, UK; 4 Neurocognitive Development Unit, University of Western Australia, Perth, Australia

## Abstract

**Background:**

We examined development of auditory temporal integration and inhibition by assessing electrophysiological responses to tone pairs separated by interstimulus intervals (ISIs) of 25, 50, 100, 200, 400, and 800 ms in 28 children aged 7 to 9 years, and 15 adults.

**Results:**

In adults a distinct neural response was elicited to tones presented at ISIs of 25 ms or longer, whereas in children this was only seen in response to tones presented at ISIs above 100 ms. In adults, late N1 amplitude was larger for the second tone of the tone pair when separated by ISIs as short as 100 ms, consistent with the perceptual integration of successive stimuli within the temporal window of integration. In contrast, children showed enhanced negativity only when tone pairs were separated by ISIs of 200 ms. In children, the amplitude of the P1 component was attenuated at ISIs below 200 ms, consistent with a refractory process.

**Conclusions:**

These results indicate that adults integrate sequential auditory information into smaller temporal segments than children. These results suggest that there are marked maturational changes from childhood to adulthood in the perceptual processes underpinning the grouping of incoming auditory sensory information, and that electrophysiological measures provide a sensitive, non-invasive method allowing further examination of these changes.

## Background

The maturation of auditory processing abilities shows a protracted developmental time course, with many specific processes not reaching adult levels until adolescence. This prolonged maturation has been shown with behavioral measures of performance on auditory tasks and by analysis of the electrical activity of the brain's response to auditorily presented information [1]. Auditory temporal processing has been a focus of particular interest because it appears important for language and literacy acquisition [2]. Behaviorally, temporal acuity has often been assessed using gap detection tasks, where participants are required to respond to brief gaps of varying duration embedded in the signal. Several studies have shown improvements in gap detection with age, but there are marked differences in the estimates of the gap detection thresholds and the sensitivity of the tasks to development, depending on the characteristics of the signal in which the gaps are embedded [3]. When the gap is embedded between 2 ms tone pips of identical frequencies, young children are able to detect gaps at thresholds of 5.6 ms [4], whereas higher thresholds are reported when the gaps are embedded in tones of differing frequencies [5,6]. Other tasks assessing auditory temporal processing, such as detection of frequency modulation and sensitivity to backward masking, show a protracted developmental course, with school-aged children performing below adult levels [7,8].

The study of auditory maturation using ERPs is complicated by the fact that there are marked age effects on the morphology, amplitudes, and latencies of the obligatory sensory components elicited by auditory stimuli. In children younger than 10 years of age, the auditory evoked response to clicks or tones is dominated by a positive peak at a latency of approximately 100 ms (P1). At approximately 10 years of age, the first negative deflection (N1) becomes apparent in the ERP and there is a corresponding decrease in P1 latency and amplitude [9-11]. Ponton et al. [10] argued, based on the developmental trajectories of the amplitudes, latencies, and dipole sources identified for these two peaks, that the modulation of the ERP waveform morphology reflected the superimposition of a developing N1 source generated in superficial cortical layers of the supratemporal gyrus.

When two auditory stimuli are presented in succession, there are three potential effects we might expect to see in the ERP: summation of responses, attenuation of the response to the second stimulus, or enhancement of the response to the second stimulus. The simplest situation is where identical ERPs are elicited to each stimulus, and these summate. In such a case there is no influence of one evoked response on the other, but the resulting waveform may look very different to that for a single tone if the time interval is short, so that the response to the second stimulus begins before the response to the first stimulus is complete. If the response to the paired-tone sequence was the result of simple summation, alignment and subtraction of the response to the single stimulus from the waveform elicited by the paired-stimulus waveform would result in an identical response to that seen to stimulus one alone.

A second possibility is that the response to the second stimulus in a pair or sequence may be attenuated relative to the first stimulus. Such attenuation has been described for responses to auditory stimulus sequences, but there is debate as to the underlying mechanism. One proposition is that attenuation is the consequence of neural refractoriness, a phenomenon well-understood at the level of individual neurons, which show reduced sensitivity to incoming stimuli immediately after firing. The notion of refractoriness can be scaled up to postulate refractory periods affecting the neural generators of the auditory ERP [12-15]. However, the notion of the refractory period has been questioned because the time course of response attenuation is not as expected. Refractoriness is greatest immediately after a neuron has responded, with sensitivity gradually increasing as time passes. However, attenuation of the response to the second stimulus in a tone pair or tone train is greater when the tones of the pair or train are separated by 400 ms than when they are separated by shorter ISIs of 100-300 ms. To account for this, Sable et al. [16] proposed a process of latent inhibition, derived from a physiological model developed by Loveless and colleagues [17,18] to explain the effects of within-pair ISI on the neuromagnetic response to tone pairs. According to this model, the initial tone of the pair activates N1 generators and this activation then spreads to other areas where further excitatory and inhibitory circuits are activated. The inhibitory circuits act in a feedback loop that reduces the activation of the N1 generators to subsequent stimuli, although this process takes approximately 400 ms to become fully functional.

The third possibility, of response enhancement to the second tone of a pair, was also discussed by Loveless et al. [17], with regard to situations where the second tone occurred after a short ISI. According to the latent inhibition model, we would not expect response attenuation if the ISI was less than 400 ms. Loveless et al. [17] reported that in this case enhancement of the response to the second tone was observed. They identified two distinct neural generators contributing to the neuromagnetic response (N100 m) and the amplitude enhancement seen at ISIs from 70-300 ms reflected activation of the later, more anteriorly-located source (N100 m^A^).

Sable et al. [16] examined amplitude modulation of the electrophysiological analogue of this neuromagnetic response (N1) following presentation of short trains of stimuli at ISIs of 100, 200, 300, and 400 ms to evaluate whether latent inhibition, refractoriness, or a combination of both processes best explained the N1 amplitude modulation. They found that N1 amplitude attenuation to the second tone of the sequence was maximal at ISIs of 400 ms, consistent with the latent inhibition model. However, they did not find enhancement of N1, compared to the first tone in the train, at ISIs less than 400 ms. These differences may reflect the use of short, five-tone trains of stimuli by Sable et al., rather than longer trains of paired-tone sequences, as used by Loveless et al. [17] and Müller et al. [3]. Although they differ in terms of whether simple summation or enhancement occurs at ISIs of less than 400 ms, Müller et al. [3] and Sable et al. [16], note that the period prior to which response attenuation is seen may correspond to the temporal window over which successive auditory events are integrated as a single unit.

According to this account, development of auditory temporal integration can be studied by measuring the brain's response to tones separated by varying ISIs. Bishop and McArthur [19] examined the neural processing of unattended auditory stimuli by comparing the event-related potentials (ERPs) elicited to single tones with those of tones pairs separated by ISIs of 20, 50, and 150 ms. Their results indicated that the ERPs elicited by tones separated by 50 and 150 ms were distinguishable in the older participants aged between 14 and 19 years. In contrast, younger participants (aged 11-13 years) failed to elicit distinguishable neural responses to tone pairs separated by intervals of 20 or 50 ms. Rather a single neural response was elicited, similar to that seen when only one tone was presented. Delays in the maturation of this discrimination process have been linked to developmental disorders of language, and these authors reported that the neural processing of auditory stimuli in a group of children with specific language impairment resembled that of the younger control cohort. The mechanisms underpinning the normal developmental trajectory are not understood, but measurement of the neural activity elicited in response to auditory stimuli presented at varying ISIs allows examination of the processes contributing to this aspect of auditory processing maturation.

The study by Bishop and McArthur [19] included only eight typically-developing children in each of two broad age bands. Furthermore, the stimuli in their tone pairs differed in frequency and duration, and they did not attempt to distinguish a summation account of responses to tone pairs from hypotheses involving refractoriness or enhancement. The present study took this line of work further. We assessed a sample of younger children than those reported in Bishop and McArthur [19] and the auditory task included a broader range of ISIs (25, 50, 100, 200, 400, and 800 ms) to allow examination of refractory processes, temporal integration, and latent inhibition. In the present study both tones of the pair were the same frequency (1000 Hz) and duration (20 ms), making it possible to compare responses to tone 1 and tone 2 directly. Tones were presented at a lower intensity than was used by Bishop and McArthur [19], and no frequency-deviant stimuli were presented.

**Figure 1:**
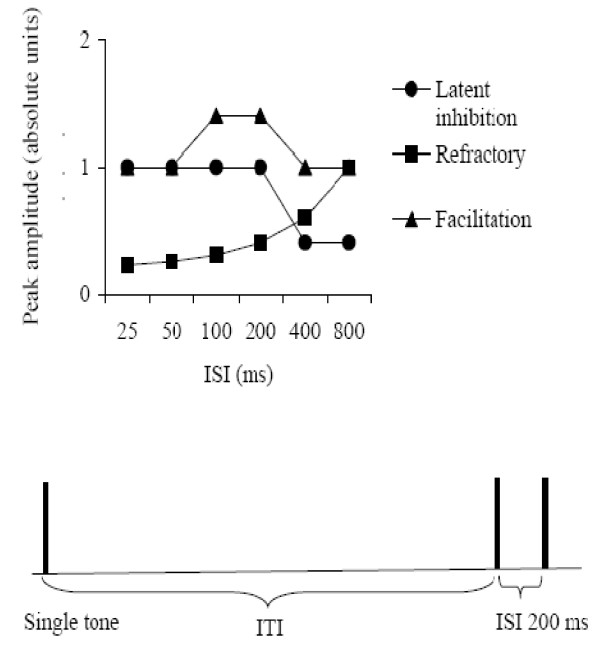
**Predicted effects on ERP mean amplitudes**. Predicted effects of the amplitude modulation expected on the basis of latent inhibition, with attenuation at ISIs of 400 ms or longer; refractoriness, where attenuation is expected to be most pronounced at the shortest ISIs and amplitude increase expected with increasing ISI; or facilitation, where enhancement of the amplitude at ISIs of 100-200 ms would be expected. No assumptions about the mutual exclusivity of these processes have been made, and the pattern of modulation could conceivably involve combinations of the profiles depicted. The lower panel of the figure presents a schematised display showing the timing for two consecutive trials, highlighting the temporal relationship between stimulus duration, the interstimulus interval between the tone pairs, and the intertribal interval.

The aims of the study were twofold: the first aim was to identify an index of integration of rapidly presented auditory information, reflected in the evoked responses to tone pairs presented at increasing ISIs. It was predicted that adults would show N1 enhancement to the second tone of the pair at within-pair ISIs of 100-200 ms relative to that elicited by the single tone. The second aim was to examine whether children would show attenuation of the early sensory ERP peak (P1) to the second tone in a pair at shorter ISIs, in line with a refractory process, or latent inhibition as proposed by Sable et al. (2004). The patterns of ERP peak amplitude modulation expected based on models of latent inhibition, refractoriness, and facilitation, across the differing ISIs used in this study, are shown graphically in Figure 1.

## Results

**Figure 2:**
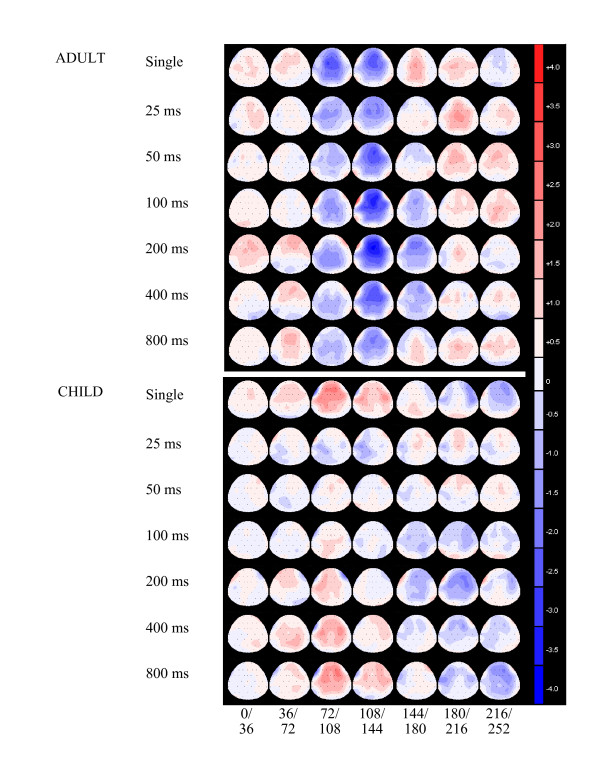
**Topographic maps of ERP amplitudes**. Topography of the ERP was first inspected to determine which electrodes to focus on. Topographic maps showing the amplitude distribution elicited following presentation of the single and second tone based on the grand average waveforms calculated for the adult sample (upper panel) and the child sample (lower panel). The average amplitude from 0-252 ms is displayed in 36 ms time intervals: 0-36 ms, 36-72 ms, 72-108 ms, 108-144 ms, 144-180, 180-216 ms, 216-252 ms. Blue shading indicates negative amplitude with respect to the pre-stimulus baseline and red shading indicates positive amplitude with respect to the pre-stimulus baseline, with maximal scaling between -4 μV to 4 μV.

Figure 2 shows the distribution across the scalp of the average amplitude during the first 200 ms following presentation of the single tone and the second tone of the pair, corrected for response overlap. Visual inspection of the scalp topography indicates that the distribution of the N1 in adults and the P1 in children were maximal at fronto-central sites, therefore the intra-class correlation coefficient was assessed at Fz in subsequent analyses.

**Figure 3:**
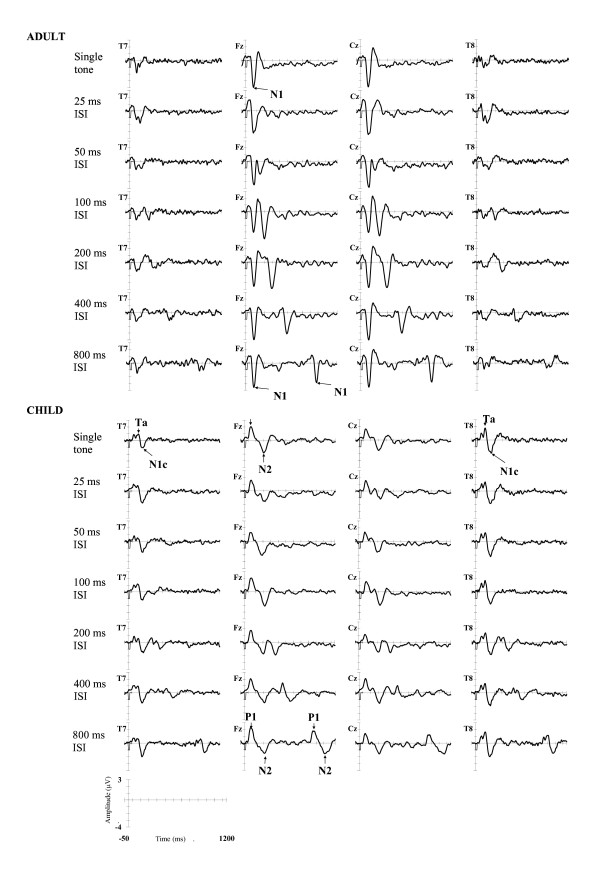
**Grand-averaged ERPs synchronised with onset of first tone**. Grand averaged ERPs at Fz, Cz, T7 and T8 elicited following the single tone and tone pair conditions at each of the ISIs. ERPs for the adult sample are displayed in the upper panel of the figure and ERPs for the child sample are displayed in the lower panel of the figure. Amplitudes (in microvolts) are represented on the y-axis. Negative voltages are plotted as a downward deflection. The x-axis represents time (in ms) from 50 ms prior to the onset of the first tone in the pair to 1200 ms post-stimulus onset. The latencies of tone onset and duration are also depicted on the x-axis.

Figure 3 shows the grand-averaged waveforms at midline (Fz, Cz) and lateral sites (T7, T8) for the adult and the child samples. Adults showed a clear N1 peak to the first tone in the pair, with a smaller amplitude lateral response also seen at sites T7 and T8. A distinct neural response to the second tone of the pair was also observed at ISIs exceeding 25 ms, although identification of the N1 peaks to the second tone was obscured by overlapping responses to the first tone. In contrast, the children's auditory evoked response was dominated by frontally-distributed P1 and N2 peaks, and the temporally-distributed T-complex can be seen at T7 and T8 sites. In children, a distinct neural response to the second tone in the pair was observed only at ISIs greater than 200 ms.

### Intra-class correlation coefficient (ICC) analyses

The similarity of the ERP waveforms elicited to the tone pairs presented at each ISI was compared to that elicited by a single tone by computing the individual ICCs over the 0-400 ms time range (see Methods), and the mean ICCs for the two age groups are summarised in Table 1. This method of analysis allowed examination of the early ERP waveforms elicited, and is particularly useful when characteristic peaks are not identifiable [20]. A high ICC indicates that the waveform to the tone pair is similar to the waveform for a single tone, i.e. that the responses to the two tones in the pair are integrated. The auditory evoked responses elicited in the child sample were considerably noisier than those elicited in the adult sample, reflected in a lower ICC between the waveform elicited to the single tone and the waveform elicited to the 400 ms ISI tone-pair condition (*t*(39) = 2.32, *p *= .03). Given this overall age group difference, the effects of ISI were analysed within each age group. For the adult sample, the main effect of ISI was significant, (*F*(4,56) = 5.78, *p *= .002, ε = .783, partial η^2 ^= .29); the ICCs were significantly lower than the 400-ms ISI condition for tone pairs presented at all ISIs below 400 ms, and in excess of 29% of the variance in scores within each condition was explained by ISI. For the child sample, there was a significant main effect of ISI, *F*(4,100) = 3.19, *p *= .03, ε = .72, partial η^2 ^= .11; ICCs were significantly lower than the 400-ms ISI condition for tone pairs presented at 200 ms ISI, but effect sizes were negligible at ISIs smaller than 200 ms (see Table 1). In sum, the waveforms of the adults indicated differentiation of the neural responses to rapid tone pairs and those to single tones, whereas for the children, this differentiation was not seen with ISIs below 200 ms.

**Table 1:** Mean Fisher's z-transformed ICC computed over the 0-400 ms latency interval, between the single tone and tone-pair conditions presented at increasing ISIs for the adult and child samples.

ISI	ICC (Adult sample)	**partial η**^**2 **^**for contrast with 400 ms ISI**	ICC (Child sample)	**partial η**^**2 **^**for contrast with 400 ms ISI**
25 ms	0.54* (0.10)	.34	0.44 (0.07)	.04
50 ms	0.44* (0.07)	.56	0.37 (0.09)	.09
100 ms	0.56* (0.06)	.39	0.52 (0.08)	.00
200 ms	0.53* (0.06)	.40	0.26* (0.09)	.41
400 ms	0.86 (0.13)		0.53 (0.08)	

* Significantly different from 400 ms ISI condition (Bonferroni-corrected α < .05/4)

Within-group standard errors are presented in parentheses.

### Analyses of ERPs corrected for response overlap

**Figure 4:**
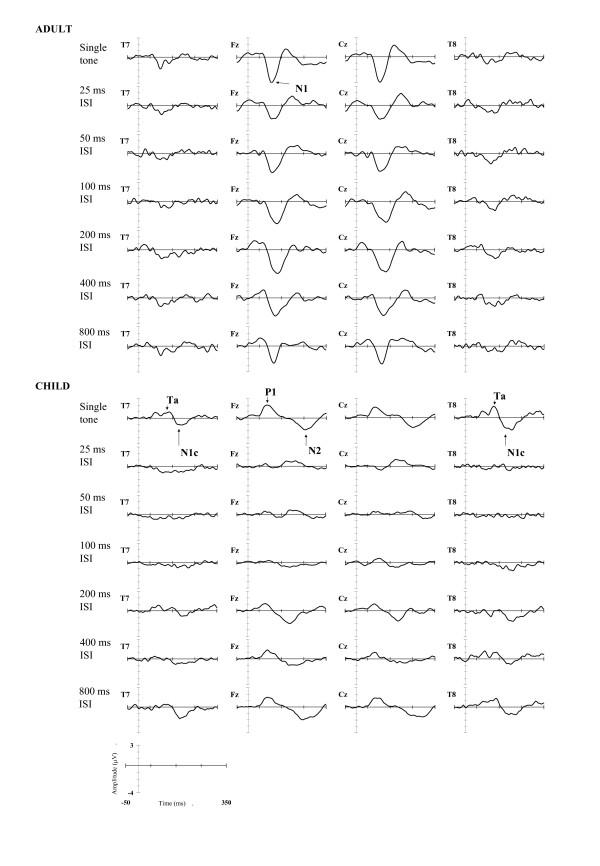
**Grand-averaged ERPs synchronised with onset of second tone**. Grand averaged ERPs at Fz, Cz, T7, and T8 elicited to the second tone in the pair, corrected for response overlap by subtraction of the ERP to the single tone. The x-axis represents time (in ms) from 50 ms prior to the onset of the second tone in the pair to 400 ms post-stimulus, with tic marks at 100 ms intervals from the start of the epoch. The y-axis represents the voltage with reference to a linked mastoid reference (in microvolts), with negative voltages plotted as a downward deflection. Grand-averaged waveforms for the adult sample are shown in the upper panel and grand-averaged waveforms for the child sample are shown in the lower panel of the figure.

When the tones were presented at short ISIs, the identification of the evoked response to the second tone of the tone pair is confounded by overlapping potentials elicited in response to the first tone. Therefore, the second set of analyses was conducted on the ERP waveforms corrected for response overlap as described in Methods. The corrected grand-averaged waveforms elicited in response to the second tone of the tone-pair are shown in Figure 4. For the adult sample, a clear N1 peak is identifiable in each ISI condition and the amplitude of the N1 peak appears smaller following the 800 ms ISI than following shorter ISIs. For the child sample, the grand-averaged waveform at midline sites is dominated by a positive deflection, peaking at 86 ms (P1), and a late negative deflection, peaking at 254 ms (N2) At temporal sites the waveform is dominated by a positive deflection, peaking at 126 ms (Ta), and a subsequent negativity, peaking at 206 ms (N1c).

### Principal Components Analysis (PCA) to identify latency ranges

**Figure 5:**
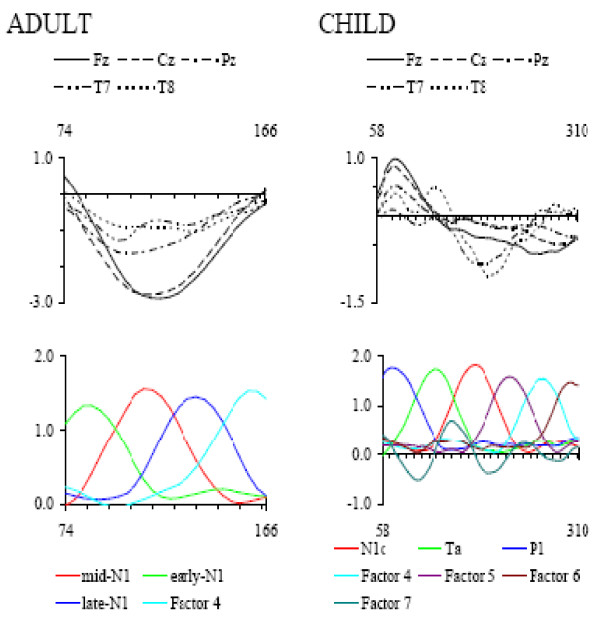
**Principal Components Analysis: factor loadings**. The upper panel of each figure displays the grand averaged ERPs at the three midline (Fz, Cz, Pz) and lateral (T7, T8) sites, summed across ISI conditions, for the interval of the ERP waveform submitted to PCA (74-166 ms in adults; 58-310 ms in children). Plots of Varimax rotated factor loadings obtained with temporal PCA of individual averaged ERP waveforms are presented in the lower panel of each figure. Data for the adult sample are presented in the left side of the figure, and for the child sample are presented in the right side of the figure.

PCA of the subtracted waveforms from adults over the interval 78 - 166 ms was conducted based on the latency ranges over which the N1 deflection differed significantly from zero, using the correction for multiple comparisons as recommended by Guthrie & Buchwald [21]. Four factors with eigenvalues greater than 1.0 were extracted, accounting for 92.7% of the variance in the data. Plots of the rotated component loadings are presented in Figure 5, graphically depicting the time course of each factor, as recommended by Picton et al. [22]. For ease of visual comparison, the grand averaged ERPs at each site, summed across participants and ISIs, are also presented on the same time-scale. The latency intervals determined for the PCA components were calculated as the interval over which the rotated factor loading for a specific component exceeded the loadings on any of the orthogonal components. The maximal loadings on the first PCA component (eigenvalue = 32.4, explaining 52.1% of the variance in the data), from 98-122 ms, corresponded in latency with the middle portion of the N1 peak in adults (labelled mid-N1). The maximal loadings on the second PCA component (eigenvalue = 14.0, explaining 22.4% of the variance in the data), from 74-94 ms, corresponded in latency with the early portion of the N1 peak (labelled early-N1). The maximal loadings on the third PCA component (eigenvalue = 8.4, explaining 13.6% of the variance in the data), from 126 ms to 146 ms, corresponded in latency with the late portion of the N1 peak (labelled late-N1). The fourth component (eigenvalue = 2.9) explained less than 10% of the variance in the data and has not been interpreted further. Based on these results, the early-N1, mid-N1, and late-N1 components in the adult sample were quantified at Fz and Cz over the latency windows 74-94 ms, 98-122 ms, and 126-146 ms respectively, for subsequent analyses. In the child sample, PCA of the corrected waveforms over the interval 58-310 ms (based on the interval over which the amplitude of the waveform differed from zero, as above) identified seven factors with eigenvalues greater than 1.0, explaining 91.5% of the variance in the data, although components 4 to 7 explained less than 10% of the variance in the data and have not been interpreted further. The maximal loadings on the first PCA component (eigenvalue = 92.6, explaining 42.2% of the variance in the data) occurred over the interval 150-202 ms, corresponding in latency to a negative deflection, seen most clearly at the lateral sites, T7 and T8. We have labeled this PCA component N1c, after the nomenclature used by Bruneau et al. [23], Pang and Taylor [24], and Woods [25]. The second PCA component (eigenvalue = 33.0, explaining 15.0% of the variance in the data) loaded maximally over the latency interval 102-146 ms and corresponds to the positive deflection at the lateral temporal sites, predominant over the right hemisphere (T8). We labelled this PCA component Ta, based on the nomenclature adopted by Woods [25]. The third PCA component (eigenvalue = 28.6, explaining 13.0% of the variance in the data), with maximal factor loadings from 58-98 ms, corresponded in latency with the clearly identifiable frontally-distributed positive peak, labelled P1. Based on the results from this analysis, the ERP components were quantified at Fz, Cz, T7, and T8 as the mean amplitudes over the latency windows 58-98 ms (P1), 102-146 ms (Ta) and 150-202 ms (N1c).

Given the marked differences in morphology of the waveforms for the adult and child sample, we have not attempted to directly relate the component structure for the adult fronto-central N1 to those identified in the analysis of the P1/T-complex observed in the child sample. Although source dipole analysis has identified similar neural generators in children and adults, it is not clear which deflections in the two waveforms represent activity from the same underlying neural generators (Albrecht et al., 2000).

### Analyses of amplitude measures of ERPs corrected for response overlap

Mean amplitudes over the early, middle, and late N1 latency intervals for the adult sample are summarized in Table 2. Mean amplitudes over the P1 and Ta latency intervals for the child sample are summarized in Table 3.

**Table 2:** Mean ERP component amplitudes, averaged across Fz and Cz, over the N1 latency range in adults (SE in parentheses) elicited to the single tone and the second tone in the tone pair, based on the ERP waveforms corrected for response overlap. Partial η^2 ^for the contrast of the amplitude at each ISI with the single tone is also shown.

ISI	N1-early	**partial η**^**2**^	N1-mid	**partial η**^**2**^	N1-late	**partial η**^**2**^
Single	-2.31 (0.45)		-3.49 (0.39)		-0.82 (0.40)	
25 ms	-1.17 (0.34)	.21	-2.06 (0.33)	.30	-1.20 (0.42)	.03
50 ms	-1.25 (0.57)	.15	-2.73 (0.60)	.08	-1.78 (0.42)	.14
100 ms	-1.38 (0.36)	.22	-2.87 (0.51)	.12	-2.96* (0.50)	.41
200 ms	-0.89 (0.28)	.35	-3.06 (0.50)	.06	-3.31* (0.45)	.66
400 ms	-0.53* (0.42)	.44	-2.45 (0.29)	.36	-2.08* (0.40)	.47
800 ms	-0.62* (0.37)	.51	-2.41 (0.37)	.34	-0.31 (0.52)	.15

* Significantly different from single tone condition (Bonferroni-corrected α < .05/6)

**Table 3:** Mean amplitudes, averaged across Fz, Cz, T7, and T8, for P1 and Ta components in children (SE in parentheses) elicited to the single tone and the second tone in the tone pair, based on the ERP waveforms corrected for response overlap.

ISI	P1	**partial η**^**2**^	Ta	**partial η**^**2**^
Single	1.11 (0.19)		1.00 (0.21)	
25 ms	-0.30* (0.29)	0.33	-0.24* (0.26)	0.32
50 ms	-0.15* (0.26)	0.37	-0.21* (0.23)	0.37
100 ms	0.21* (0.21)	0.43	-0.03* (0.27)	0.33
200 ms	0.58 (0.23)	0.10	-0.19* (0.24)	0.41
400 ms	0.73 (0.19)	0.10	0.43 (0.24)	0.13
800 ms	0.89 (0.27)	0.02	0.82 (0.20)	0.01

* Significantly different from single tone condition (Bonferroni-corrected α < .05/6)

Partial η^2 ^for the contrast of the amplitude at each ISI with the single tone is also shown.

For adults, early-N1 elicited by the second tone of the pair was significantly smaller in amplitude than following a single tone at longer ISIs, with the amplitude attenuation failing to reach statistical significance at ISIs below 400 ms (main effect of ISI, *F*(6, 84) = 2.50, *p *= .028, ε = .64, partial η^2 ^= .15). This agrees with findings by Sable et al. [19] for brief trains of tones, and is consistent with attenuation resulting from an inhibitory process that does not become fully functional for approximately 400 ms after the first tone onset. Amplitude modulation of the mid-N1 latency interval as a function of ISI failed to reach statistical significance (*F*(6, 84) = 1.24, *p *= .30, ε = .56, partial η^2 ^= .08). In contrast, amplitude over the late-N1 latency interval was significantly enhanced to the second tone, relative to the single tone, when separated by ISIs of 100-400 ms (main effect of ISI *F*(6, 84) = 6.37, *p *= .001, ε = .52, partial η^2 ^= .31).

In the child sample, P1 amplitude elicited to the second tone of the pair was significantly reduced when the second tone was presented at shorter ISIs, but not at longer ISIs, consistent with attenuation resulting from a refractory effect rather than an inhibitory process. P1 amplitude was significantly reduced, compared to the single tone, when tone pairs were presented at ISIs shorter than 200 ms (main effect of ISI, *F*(6, 150) = 5.39, *p *< .001, ε = .76, partial η^2 ^= 0.18). To assess whether P1 was elicited at the shorter ISIs, the amplitude of the waveform over the P1 latency range at Fz was compared with a test value of zero. No statistically significant P1 was elicited at 25 ms ISIs (*t*(25) = -0.23, *p *= .82), 50 ms ISIs (*t*(25) = 0.47, *p *= .64), or 100 ms ISIs (*t*(25) = 1.19, *p *= .25). Ta amplitude was more positive over the right hemisphere (T8) than the left hemisphere (T7) (main effect of site, *F*(3,75) = 3.30, *p *= .04, ε = .73, partial η^2 ^= 0.12), and was significantly less positive for tones presented at ISIs shorter than 400 ms than for the single tone (main effect of ISI, *F*(6,150) = 5.49, *p *< .001, ε = .78, partial η^2 ^= 0.18).

**Figure 6:**
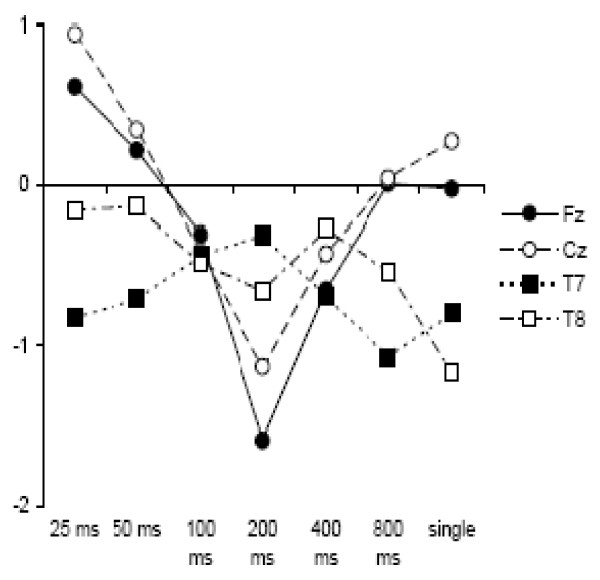
**N1c mean amplitudes in child sample**. Mean amplitudes over the N1c latency range at each ISI for the child sample, illustrating the statistically significant ISI x site interaction.

ISI modulated N1c amplitude, and the effect of ISI was further qualified by a significant interaction with site (*F*(18,450) = 4.91, *p *< .001, ε = .48, partial η^2 ^= 0.16). Separate analyses of the effect of ISI were conducted at each site, adjusting for the number of comparisons. Thus, the alpha level used for each test of significance was 0.0125. The mean amplitudes at each site across the differing ISI conditions are presented graphically in Figure 6. The amplitude over this latency interval was significantly enhanced at 200 ms ISIs relative to the single tone condition at Fz and Cz sites, with statistically non-significant amplitude modulation at the lateral sites, T7 and T8 (Fz main effect of ISI, *F*(6,150) = 4.61, *p *< .001, partial η^2 ^= 0.16; Cz main effect of ISI, *F*(6,150) = 3.88, *p *= .001; partial η^2 ^= 0.13; T7 main effect of ISI, *F*(6,150) = 1.41, *p *= .23, partial η^2 ^= 0.05; T8 main effect of ISI, *F*(6,150) = 1.91, *p *= .08, partial η^2 ^= 0.07). This result indicates that the fronto-centrally distributed negative enhancement over this interval is distinct from the temporally-distributed N1c component. In view of the latency of the component (150-202 ms), the topography of the ISI effect (fronto-central), and the effect of the experimental manipulation of ISI (amplitude enhancement), we have identified this component as equivalent to the late-N1 identified in the adult sample.

## Discussion

The primary aim of the current study was to examine the development of auditory processing, as indexed by electrophysiological measures which are not influenced by response demands that can contribute to age-related differences on behavioral measures. The results from this study showed that there is a marked change in the neural responses to brief tone pairs from childhood to adulthood. In children a distinct neural response was elicited at ISIs above 200 ms, but not when tone pairs were separated by ISIs of 25 ms, 50 ms, or 100 ms, whereas in early adulthood, a distinct neural response was elicited to tones presented at ISIs of 25 ms and longer. The results provide further validation for the use of the ICC in the study of auditory temporal processing [19] and extend the previously reported findings by showing that this measure can provide a sensitive index of neural responsiveness to rapidly presented auditory information in children as young as 7 years. It is necessary to be cautious when directly comparing the results from the present study with those reported by Bishop and McArthur given the methodological differences between the two studies, such as differences in the specific ISIs used; differences in the duration and intensity of the tones, and differences in the tasks that children were engaged in during tone presentation. Nevertheless, the observed estimate of between 100 and 200 ms for the younger children (7-9 years) in our study and between 50 and 150 ms for the older children (11-13 years) reported in the earlier study [19] leads us to speculate that developmental changes in auditory temporal processing may be identifiable over these age ranges with the ICC index, and warrants further investigation using this technique. However, it is noted that the signal-to-noise ratio was lower in the child waveforms than in the adult waveforms, and this factor may have contributed to the attenuation of the response to the second tone at ISIs of 25-100 ms.

The absolute magnitude of the temporal resolution in children estimated in the present study and Bishop et al. [19] is considerably larger (between 50 and 200 ms) than with previous estimates using behavioral gap detection tasks (between 3 and 40 ms) [4-6,26,27]. In adults, the elicitation of the P1-N1-P2 complex has been shown to relate to behaviorally determined gap detection thresholds, with attenuation of the response to the second tone when presented at subthreshold gap durations [28]. It does not therefore seem appropriate to regard the developmental trend seen here as indicative of an improvement in auditory temporal discrimination. An alternative way of interpreting the results is to see the distinctive neural responses to tones at differing ISIs as reflecting a process of temporal integration, whereby repeated sounds are grouped together in a single auditory object. The notion of auditory objects is a highly disputed one, and the specific features, such as the spectral and temporal composition of the incoming sound, which define an object have been the subject of recent neuroscientific research [29]. The results from the present study highlight the importance of the contextual temporal features of the incoming sound source in auditory perception, as postulated by Krumbholz et al[30], and show that detailed analysis of the N1 sub-components that can be identified in electroencephalograph (EEG) [31] and magnetoencephalograph (MEG) recordings [32] can contribute to an understanding of the role of temporal processing in auditory perception. A listener may still be able to discriminate changes in stimulus features of an auditory object, as may be caused by presence of a gap [5], and so this explanation is compatible with gap detection thresholds being smaller than auditory integration thresholds. An explanation in terms of auditory integration was proposed by Loveless [17] following observation of an enhancement in the neuromagnetic response (N100 m^A^) to tone pairs presented at stimulus onset asynchronies ranging from 70-300 ms. However, again, the time intervals obtained in this study are not entirely in line with expectation. Wang et al. [15], for instance, compared children and adult's neural responses to deviant tones in a mismatch negativity (MMN) paradigm, and noted that a sequence of two different deviants elicited a single MMN when tones were separated by inter-stimulus intervals of 100 ms for adults, 200 ms for 9- to 11-year-olds, and 300 ms for 5- to 8-year-olds. If we regard the enhancement of the fronto-central negativity culminating at approximately 145 ms in adults and 170 ms in children as a marker of the integration of the second stimulus with the preceding stimulus, then our results suggest a somewhat shorter window of temporal integration of 200 ms for 7-9 year-old children, and, as far as the morphology of the response to a tone pair is concerned adults show a distinctive response to the second tone with ISI as short as 25 ms. However, it should be noted that the estimates derived from the MMN paradigm indicate the earliest latency that is longer than the temporal window of integration, rather than the ISIs that fall within the temporal window of integration. An alternative explanation for the varying estimates of the temporal integration time could relate to the postulated neural mechanisms contributing to auditory integration associated with different psychoacoustic features. In the double deviant mismatch paradigm used by Wang et al. [15], the first deviant stimuli differed from standard tones in frequency and the second deviant tone differed from the standard tones in intensity. Temporal integration time was assessed by determining the ISI at which the second deviant elicited a distinct MMN. The finding of two distinct MMNs at ISIs exceeding 200 ms supports the contention that both features were independently coded despite the regular co-occurrence of the deviants in the sequence. Neural coding that contributes to auditory segregation based on frequency commences peripherally, with tonotopic representation at the cochlear level. Perceptual streaming following presentation of continuous sequences of tones increases with increases in the magnitude of the frequency difference between the tones [33]. In the present study, auditory objects were defined by temporal separation of sequential tone pairs presented at the same frequency and intensity. Estimates of the temporal integration window may depend on the specific features underpinning auditory segregation. Alternatively, differing estimates of the temporal window of integration in these two studies could relate to the refractory period of the neural generators. In the current study, tone pairs were separated by a longer inter-trial interval (two seconds), allowing greater recovery of the neural responses to stimuli than was possible with the continuous tone sequences presented at the relatively short ISIs by Wang et al. [15]. These results suggest that the notion of a constant temporal window of integration that applies across all stimulus types and methods may be misguided, and that both stimulus characteristics and methods of measurement may give different temporal estimates.

The second major aim of the present study was to examine the relative contributions of refractoriness and latent inhibition to the amplitude modulation observed at varying ISIs in adults and children. In adults, the early N1 was attenuated at longer ISIs, with the largest effect at ISIs of 800 ms, consistent with the operation of a latent inhibitory process, as suggested by Sable et al. [16]. In children, the amplitude of P1 was attenuated at shorter ISIs, consistent with a refractory process, and there was no evidence for the operation of the latent inhibitory process that had been observed at longer ISIs in the adult sample. It is not possible to determine from the current results whether the latent inhibitory process is not yet functional in 7-9 year-old children, or whether it operates over a longer temporal interval than we examined. Our results raise the possibility that one factor influencing the length of the temporal window in children is the enhanced refractoriness of neurons in auditory cortex. Given that we found the same pattern of results using two different forms of analysis (comparison of amplitudes in the subtracted waveforms, and size of the intra-class correlation coefficient for the unsubtracted waveforms), it is unlikely that they are due to artefact introduced by the subtraction method. It is possible that the suppression of P1 at 100 ms ISIs observed in both analyses is due to overlap with the N2 elicited in response to the previous tone, although we think this explanation does not fully explain the results, given that the N2 component had been substantially reduced by application of the 2 Hz high-pass filter, and given that we observed a similar amplitude attenuation of the Ta peak at similar ISIs at lateral sites (T7 and T8) where N2 was not evident. The results from the current study are at odds with the findings reported by Dinces and Sussman [34], where a pronounced P1, modulated by the intensity of the stimuli, was elicited at 100 ms ISIs to deviant frequency tones in 9-11 year-old children. As noted by these authors, modulation of P1 amplitude in their study may have reflected attentional capture by relatively loud stimuli embedded within constantly varying intensity stimuli and further research is required to determine whether the differing results are related to differences in the ages of the samples studied (7-9 years in the current study; 9-11 years in their study) and/or to differences in the acoustic deviance of the stimuli relative to the preceding train (same intensity for all tones in the current study, intensity deviants ranging from 66 dB to 86 dB following standard 70 dB tones in their study).

In terms of the physiological model developed by Loveless et al. [17] and McEvoy et al. [18], the enhancement of the N100 m^A ^observed at short ISIs reflects the activation of a neural generator located in auditory association areas summating with the response to successively presented auditory information. Other factors related to cortical maturation, such as synaptic efficacy, myelination and conduction velocity could also have contributed to delay activation of these association areas and alter the timing of the subsequent integration of successively presented tones, thus modulating the neural response to the second tone at faster rates.1 The results could also be interpreted in terms of a phase-resetting account, whereby the peaks and troughs in the averaged auditory ERP result from ongoing brain oscillations being synchronised at the onset of a stimulus [35]; in this case we would need to postulate that the likelihood of phase resetting is a function of the interval between two stimuli.

Although we have identified late N1 enhancement in response to auditory stimuli in the present study, Wang et al. [36] report a similar enhancement of the N1 elicited following presentation of somatosensory stimuli, raising the possibility that this effect may reflect modulation that is common across different sensory systems.

It is difficult to dissociate facilitation from inhibition in most experimental designs, and Sable et al. [16] argued that inhibitory mechanisms more parsimoniously explained the postulated facilitation observed in studies where tones had been presented at varying ISIs. By comparing the neural response to tone pairs with that elicited by a single tone, and by dissociating the underlying N1 sub-components with PCA, the results from the current study suggest that both processes operate, as proposed by McEvoy et al. [18].

## Conclusions

These results indicate that adults integrate sequential auditory information into smaller temporal segments than children, and suggest that there are marked maturational changes from childhood to adulthood in the perceptual processes underpinning the grouping of incoming auditory sensory information. In future studies, it would be valuable to include behavioral measures of auditory temporal grouping; our prediction is that this measure will relate to the ERP indices studied here. In addition, future research investigating the relationship between the electrophysiological indices elicited in the present paradigm and individual differences in language proficiency is warranted.

## Methods

### Participants

Adult sample: Fifteen healthy participants ranging in age from 19-28 years were recruited. All volunteered to take part in the experiment after receiving information regarding the nature of the procedures and provided written informed consent. None reported hearing difficulties.

Child sample: Children participated in a two-day holiday activity program, designed to investigate the cognitive, emotional, and social development of children aged 7-9 years (Project K.I.D.S.). ERP data were excluded from individuals where a history of neurological disorders was reported, or where reliable auditory evoked responses were not elicited to the tones (see section on EEG acquisition and analysis for details). Auditory ERPs from a sample of 28 children ranging in age from 7 years 2 months to 9 years 11 months were available for analyses.

### Tone stimuli

Auditory stimuli were 1000 Hz, 20 ms sinusoidal tones with 2 ms rise and fall times. In the paired-tone conditions, a second identical tone was presented following ISIs of 25 ms, 50 ms, 100 ms, 200 ms, 400 ms, or 800 ms. Sound intensity was calibrated using a 1-second continuous 75 dB SPL tone measured with a Bruel and Kjaer sound level meter.

### Procedure

An electrode cap was fitted and participants were presented with the auditory stimuli while they concurrently completed a visual flanker task and during interspersed breaks as they silently read or played electronic games. They were instructed to ignore the tone sequences, but to remain quiet and still throughout the recording session. Trials were presented in blocks at an inter-trial interval of 2 s, with random selection of each of the seven tone pairs within each block. Delivery of the first tone of the pair was randomly jittered between 300 and 800 ms from the start of the trial to avoid anticipatory ERP effects and prevent synchronization of the auditory evoked responses with the visual evoked responses elicited during the cognitive task. The number of trials varied across participants, as the task was terminated at the end of the allocated scheduled timeslot and there was variation across individuals in the time taken to apply the electrode cap. The length of the interspersed breaks between the segments of flanker task was increased for the children. For the adult sample, an average of 68 epochs were included in the individual averaged ERPs at each ISI (range 63 - 74), and for the child sample an average of 115 epochs were included in the individual averaged ERPs at each ISI (range 72 - 135).

The protocol was approved by the University of Western Australia Human Research Ethics Committee (RA/4/1/1436).

### EEG acquisition and analyses

The electroencephalogram (EEG) was recorded continuously from 33 scalp locations using an electrode cap (EasyCap, Montage 40, excluding TP9 and TP10). Electrodes were also placed above and below the left eye, and on the left and right mastoids with an averaged mastoid reference digitally computed online. The ground was located at site AFz. Data were amplified with a NuAmps 40-channel amplifier, and digitized at a sampling rate of 250 Hz. The analog signal was filtered online with a low pass 70 Hz filter and 50 Hz notch filter, and digitally filtered off-line with a 2-30 Hz, zero phase shift band-pass filter (12 dB down). The 2 Hz high-pass filter was applied to reduce overlap from slow responses to the first tone [37], and is within the range of high-pass filters applied in much of the literature cited (1 Hz to 5 Hz). Amplitude modulations of slow ERP components (e.g. N2, P3) can not be sensibly examined in the current data set, and no specific hypotheses about the effects of ISI on these components had been made. Ocular artifact reduction was performed on the continuous EEG using the algorithm developed by Semlitsch et al. [38] with regression-based subtraction of the averaged blink artefact identified in the bipolar VEOG channel. Epochs encompassing an interval from 50 ms prior to the onset of the first tone in the pair to 1200 ms post-stimulus were extracted and trials contaminated by artifact exceeding 150 μV rejected from the individual subject ERP averages.

Reliability of the individual auditory ERPs was assessed by means of the intra-class coefficient (ICC), computed over the interval from stimulus onset to 400 ms post-stimulus, between the individual's auditory evoked response to the single tone and the first tone of the tone pair presented at 800 ms ISI. One would expect these two conditions to give identical ERPs to the first tone and so they can be used to assess reliability of an individual's data.

Two methods for analysis of the ERP waveforms elicited by the tone pairs were used. In the first set of analyses, the similarity of the evoked responses to the tone pairs presented at increasing ISIs was compared by computing the ICC between the individual's single tone ERP and the ERP elicited to the tone pairs presented at increasing ISIs, consistent with the approach reported by Bishop and McArthur. The ICC provides a measure of the similarity of the waveform amplitude and shape, with higher values representing greater similarity between the ERPs elicited to the different tone-pair conditions. ICC should be low for conditions where two distinct neural responses were represented in the ERP. The ICC is a summary statistic, similar to the Pearson correlation coefficient, sensitive to differences in the magnitude of the values as well as to differences in the pattern of the numerical arrays (i.e. waveform shape). For the present analyses, the ICC between the two arrays, X and Y, containing N numbers of pairs of data points was computed as (MS between - MS within)/(MS between + MS within), where MS between = (((Σ (X^2^) + Σ (Y^2^) + 2 * Σ (X.Y))/2 - (Σ (Y^2/2 * N)))/(N-1) and MS within = (0.5 * (Σ (X^2^) + (Y^2^)) - Σ (X.Y)/N (Bishop et al., 2005). ICCs were computed over a 0-400 ms interval, normalised by applying the Fisher's z-transformation. Repeated measures ANOVA was used to assess the statistical significance of the ICC modulation across the five ISI conditions (25, 50, 100, 200, and 400). Differences in the magnitude of the ICC at each ISI were compared with the ICC of the 400 ms tone pair condition, as any response to the second tone would not have been elicited within the 0-400 ms epoch. Given the predominance of the early negative deflection (N1) in the adult waveform in contrast to the P1-N2 complex evident in the child waveform, statistical analyses were conducted within each age group. Post-hoc analyses were conducted by comparing the mean ICC at each ISI with the 400 ms ISI condition, and the overall family error rate adjusted for the number of post-hoc comparisons conducted.

In the second set of analyses, responses to the second tone of the pair were examined by correcting for response overlap, consistent with the approach reported by Sable et al. [16]. Specifically, the ERP waveform elicited following presentation of the single tone condition was subtracted from each of the paired-tone ERP averaged waveforms. The resultant corrected ERP waveform was baseline adjusted around the 50 ms preceding the onset of the second tone, and the onset of the second tone in the pair aligned as the zero time-point. To assist in the identification and quantification of the N1 component structure [14,25], temporal principal components analysis (PCA) based on the covariance matrix, with Varimax rotation of the factors [39,40] was conducted on the individual ERP averaged data, computed for each ISI condition at the midline and lateral sites (Fz, Cz, Pz, T7, T8) to identify the latency ranges over which to quantify the ERP mean amplitudes calculated relative to the mean amplitude of the 50 ms pre-stimulus epoch. To assess the reliability of the PCA components extracted, the Mahalanobis distance of each case was determined, and cases identified as multivariate outliers with respect to the solutions were removed from the dataset. Of the 525 cases entered into the PCA for the adult sample (5 scalp sites × 7 conditions × 15 participants), nine cases were identified as multivariate outliers, and excluded for the determination of the latency intervals over which the mean amplitudes were subsequently measured. Of the 980 cases entered into the PCA for the child sample, 24 were identified as multivariate outliers and similarly excluded from the PCA. Repeated measures ANOVAs were used to assess the statistical significance of the ERP mean amplitude modulation across the seven ISI conditions (single tone, 25, 50, 100, 200, 400, and 800 ms) and post-hoc analyses were conducted by comparing the amplitudes at each ISI with the amplitude elicited by the single tone. This comparison allows examination of the independent effects of facilitation and attenuation, without the confound of response overlap, which affects only the short ISI conditions.

The distributions of variables within each condition and age group were inspected for skewness and kurtosis, and univariate outliers (*z *> 3.29). Data from two children were excluded from the analyses. The overall family error rate was adjusted for the number of post-hoc comparisons conducted. To account for violations of sphericity in analyses where there were greater than two levels of the repeated measures factor, probability levels based on the Greenhouse-Geisser adjustment to the degrees of freedom are reported [41-43], together with the uncorrected degrees of freedom and epsilon.

## Authors' contributions

AMF, MA, CR and DVMB developed the experimental protocols, AMF and TS collected and averaged the ERP data; AMF and DVMB analysed the data and prepared the manuscript. All authors reviewed and approved the final manuscript.
